# AMPK-dependent autophagy upregulation serves as a survival mechanism in response to Tumor Treating Fields (TTFields)

**DOI:** 10.1038/s41419-018-1085-9

**Published:** 2018-10-19

**Authors:** Anna Shteingauz, Yaara Porat, Tali Voloshin, Rosa S. Schneiderman, Mijal Munster, Einav Zeevi, Noa Kaynan, Karnit Gotlib, Moshe Giladi, Eilon D. Kirson, Uri Weinberg, Adrian Kinzel, Yoram Palti

**Affiliations:** 1 Novocure Israel, Haifa, Israel; 2 Novocure GmbH, Luzern, Switzerland; 3 Novocure GmbH, Munich, Germany

## Abstract

Tumor Treating Fields (TTFields), an approved treatment modality for glioblastoma, are delivered via non-invasive application of low-intensity, intermediate-frequency, alternating electric fields. TTFields application leads to abnormal mitosis, aneuploidy, and increased cell granularity, which are often associated with enhancement of autophagy. In this work, we evaluated whether TTFields effected the regulation of autophagy in glioma cells. We found that autophagy is upregulated in glioma cells treated with TTFields as demonstrated by immunoblot analysis of the lipidated microtubule-associated protein light chain 3 (LC3-II). Fluorescence and transmission electron microscopy demonstrated the presence of LC3 puncta and typical autophagosome-like structures in TTFields-treated cells. Utilizing time-lapse microscopy, we found that the significant increase in the formation of LC3 puncta was specific to cells that divided during TTFields application. Evaluation of selected cell stress parameters revealed an increase in the expression of the endoplasmic reticulum (ER) stress marker GRP78 and decreased intracellular ATP levels, both of which are indicative of increased proteotoxic stress. Pathway analysis demonstrated that TTFields-induced upregulation of autophagy is dependent on AMP-activated protein kinase (AMPK) activation. Depletion of AMPK or autophagy-related protein 7 (ATG7) inhibited the upregulation of autophagy in response to TTFields, as well as sensitized cells to the treatment, suggesting that cancer cells utilize autophagy as a resistance mechanism to TTFields. Combining TTFields with the autophagy inhibitor chloroquine (CQ) resulted in a significant dose-dependent reduction in cell growth compared with either TTFields or CQ alone. These results suggest that dividing cells upregulate autophagy in response to aneuploidy and ER stress induced by TTFields, and that AMPK serves as a key regulator of this process.

## Introduction

Tumor Treating Fields (TTFields) are an established anti-mitotic treatment modality delivered via non-invasive application of low-intensity (1–3 V/cm), intermediate-frequency (100–300 kHz), alternating electric fields to the tumor region^1–3^. In a randomized phase 3 study (NCT00916409) TTFields in combination with maintenance temozolomide significantly prolonged progression-free and overall survival of newly diagnosed glioblastoma patients when compared with patients receiving maintenance temozolomide alone^4^. Previous studies have demonstrated the effectiveness of TTFields application in various cancer cell lines, as well as in in-vivo models and in the clinical setting^2,3,5–7^. TTFields intrinsically affect molecules that possess high electric dipole moment and promote a number of anti-mitotic effects including the disruption of the spindle structure through microtubules depolymerization and perturbation of cytokinesis through mitotic Septin complex mislocalization, both of which may ultimately lead to mitotic catastrophe^3,8,9^.

More recent studies have also revealed the inhibitory effects of TTFields on cell migration and invasion via downregulation of phosphoinositide 3-kinase (PI3K)/AKT/nuclear factor-κB signaling^10^ and the capability of TTFields to sensitize cancer cells to radiation by impeding the DNA damage response, possibly through downregulation of the BRCA1 signaling pathway^11–13^.

Several studies have shown that cells treated with TTFields demonstrate an increase in cell volume and granularity^9,14^. Increased cellular granularity is typically associated with senescence and autophagy^15,16^. As senescence was not detected in cells treated with TTFields, we hypothesized that the origin of the observed granularity may be due to the accumulation of autophagosome vesicles^8^. A recent study supports this hypothesis by providing evidence that TTFields induce autophagy in glioma cell lines^17^. Observations that autophagy was stimulated under stress conditions and was shown to be involved in cell survival and proliferation have prompted interest in the relevance of autophagy in human disease, including cancer, and its role in treatment resistance^18,19^. The role of autophagy in cancer is complex^20,21^. Autophagy can have a tumor suppressive function at early stages of cancer development and promote tumor cell survival in established tumors^22^. Autophagy also facilitates the resistance of tumor cells to anticancer agents^23^ and to radiation^24^.

The objective of the current work was to understand the effects of TTFields on cancer cells in terms of autophagy. Specifically, we show that the abnormal mitosis induced by TTFields upregulate proteotoxic stress response leading to AMP-activated protein kinase (AMPK) activation and increased autophagic flux in treated cells. Our findings support that the enhanced autophagy serves as a resistant mechanism to TTFields, which could be circumvented by targeting autophagy.

## Results

### Effects of TTFields on cellular granularity

**Fig. 1:**
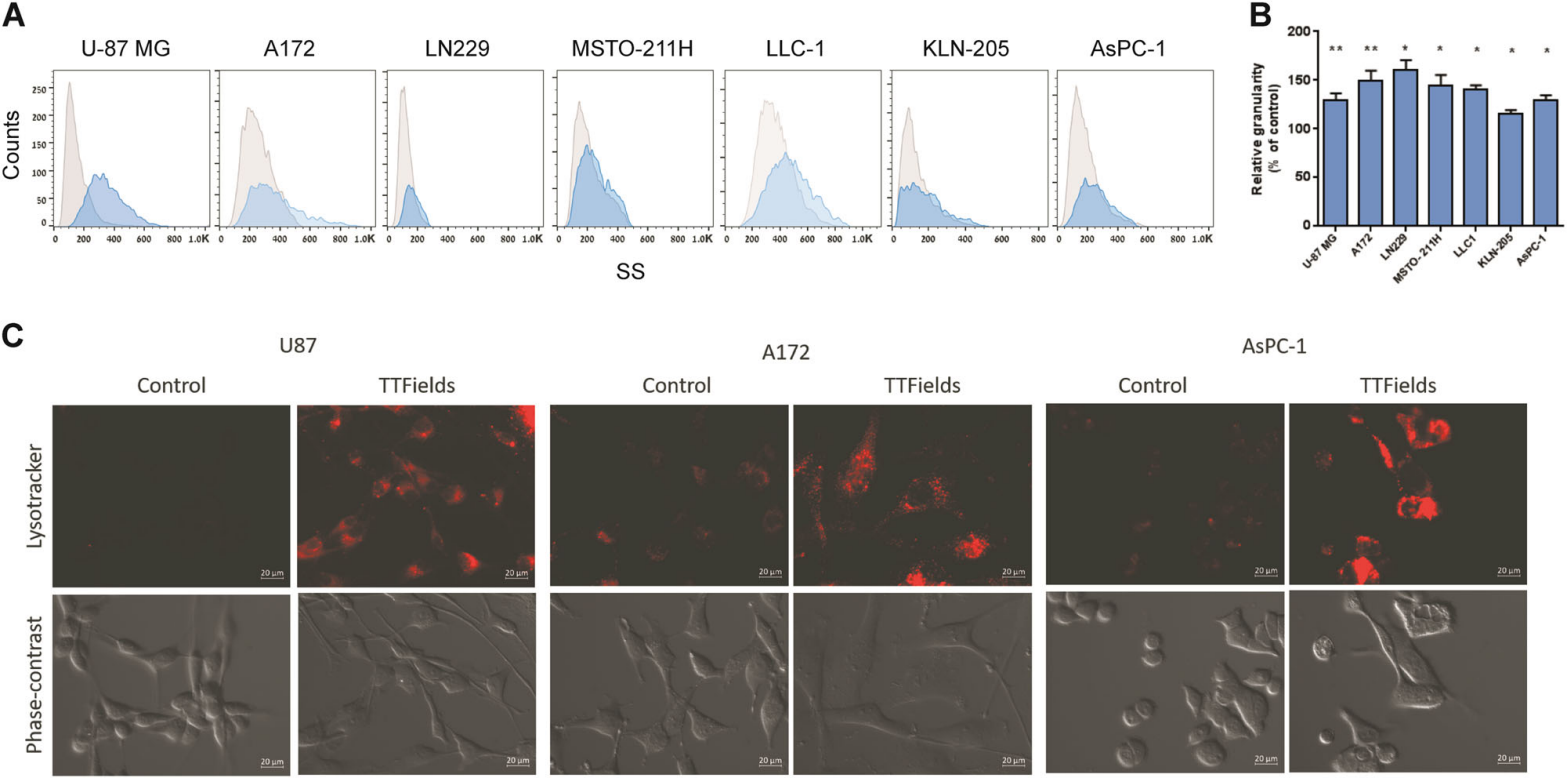
TTFields application leads to increased cellular granularity. **a**, **b** U-87 MG, A172, LN229, MSTO-211H, LLC-1, KLN-205, and AsPC-1 cancer cells were either left untreated or treated with TTFields at the optimal treatment frequency for 72 h. **a** Side-scatter parameters were measured using flow cytometry. **b** Median side-scattered values following TTFields application relative to respective control represent the change in cellular granularity (0.01 < *P < 0.05, **P < 0.01 from corresponding control group, Student’s *t*-test, *n* = 3). **c** Fluorescent microscopy images (LysoTracker staining) of U-87 MG, A172, and AsPC-1 cells (upper panels), and light microscopy (phase contrast) (lower panels), following 72 h incubation with or without TTFields application. Original magnification: × 40

To establish whether changes in cell granularity are a common outcome of TTFields application, we used flow cytometry analysis of side-scatter parameters (i.e., granularity), in various cancer cell lines, including the following: mesothelioma (MSTO-211H), glioma (U-87 MG, A172, LN229), lung (LLC-1, KLN-205), and pancreatic (AsPC-1) cancer^25^. In all cell lines tested, TTFields application resulted in changes in cellular granularity (Fig. 1a, b)^25^. This can potentially be attributed to lysosomes accumulation, which was confirmed by fluorescent microscopy of LysoTracker-stained cells, which demonstrated larger acidic lysosomal pool in TTFields-treated cells (Fig. 1c).

### TTFields increase autophagosome formation and autophagic flux

**Fig. 2:**
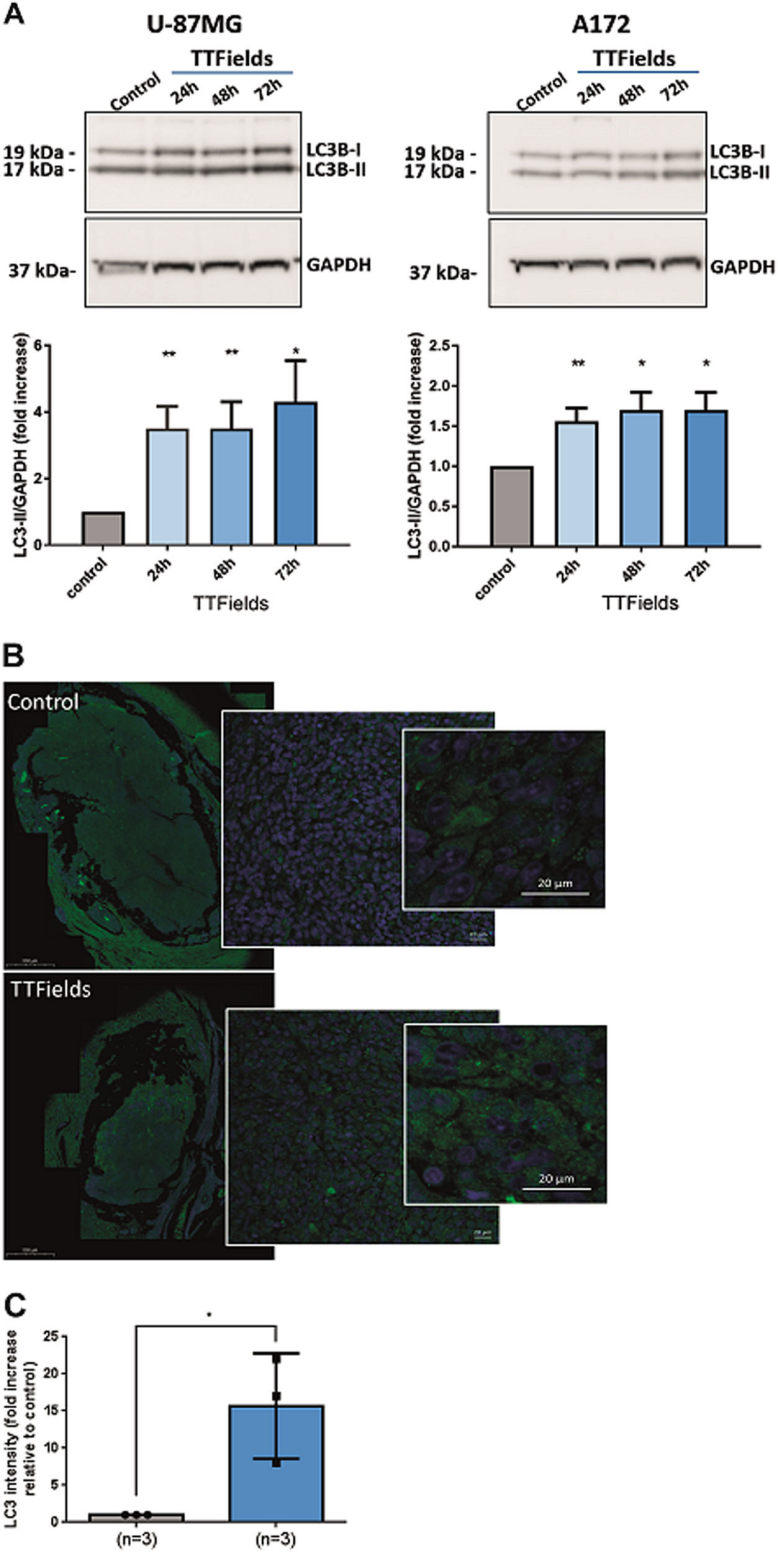
TTFields induce autophagy in glioma cell lines. **a** U-87 MG and A172 cells were either left untreated or treated with TTFields at the last 24 h, 48 h, or 72 h of culturing. All cultures were plated on the same time, incubated overnight to allow cell attachment, and collected 72 h afterwards. Cells were collected, lysed, and samples were analyzed using immunoblotting for LC3 and GAPDH. Upper panel: representative blots. Lower panel: densitometric quantification of immunoblot signal, showing an average of at least three independent experiments (0.01 < *P < 0.05, **P < 0.01, Student’s *t*-test). **b** Paraffin-embedded sections from sham- or TTFields-treated rats were stained with anti-LC3 Ab (green) and DAPI (blue). Representative images are presented. **c** Quantification of LC3 intensity, presented as fold increase from corresponding control (*P < 0.05, Student’s *t*-test)

To explore whether the appearance of the cellular lysosomes following TTFields application is attributed to autophagy, we used immunoblot assay to quantify light chain 3 (LC3) in U-87 MG and A172 glioma cell lines. LC3 is a cytoplasmic protein that on induction of autophagy is converted to LC3-II through lipidation, allowing its association to the autophagic vesicle membrane^26^. Immunoblot assay revealed that LC3-II signal increased after TTFields application, and that this increase in autophagosome formation was dependent on treatment duration (Fig. 2a). To test whether TTFields also increased autophagosomes formation in vivo, we treated Fisher rats inoculated intracranially with F98 glioma cells (as was previously reported by Kirson et al.^2^) with either TTFields or sham control, and stained the tumor sections for LC3. Application of TTFields was associated with noticeable increase in autophagy (Fig. 2b, c).

**Fig. 3:**
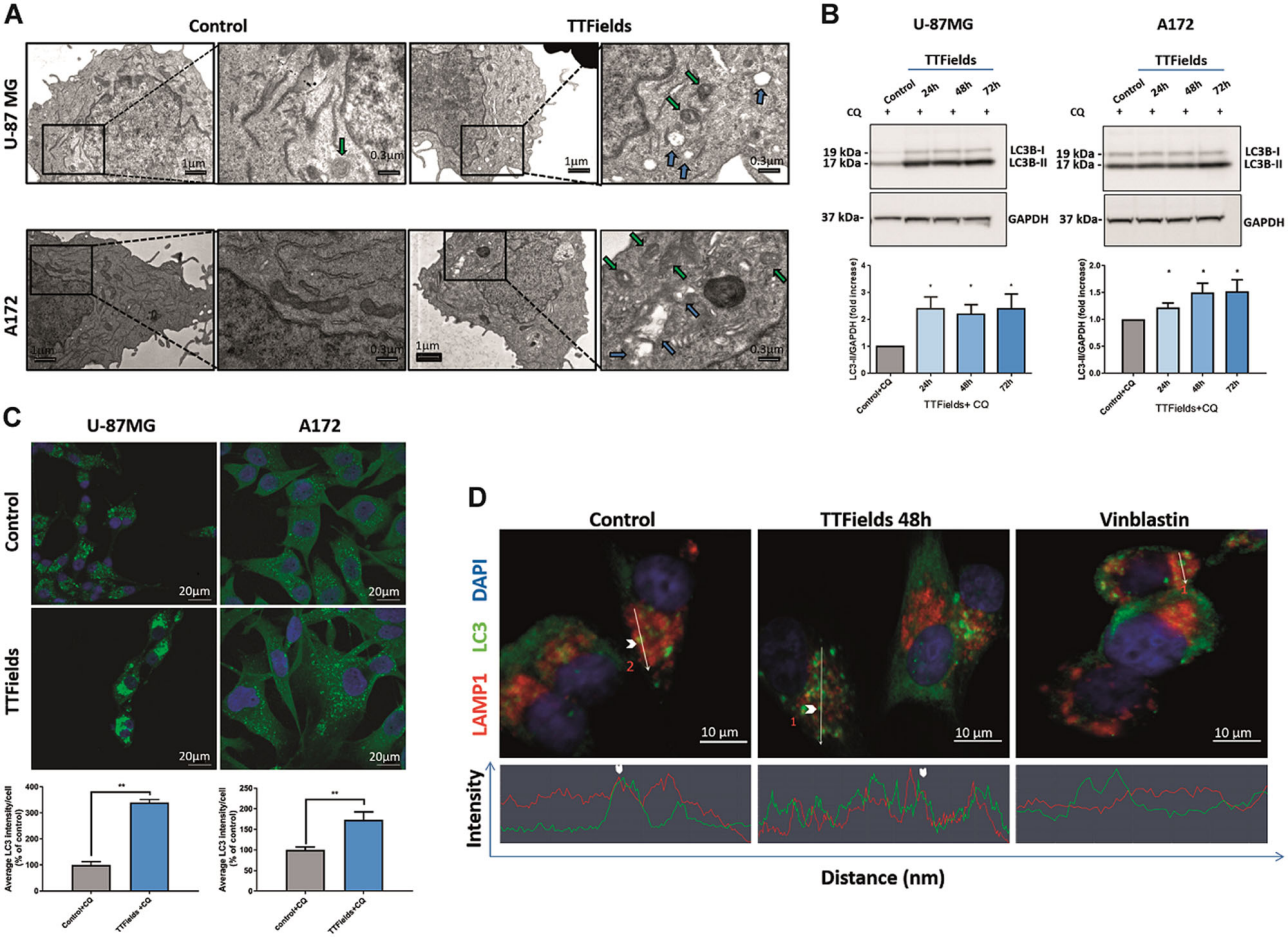
TTFields application leads to increased autophagic flux. **a** Ultra-structural STEM electron microscopy analysis of U-87 MG (upper panel) and A172 (lower panel) cells treated with TTFields for 48 h. Autophagosomes (blue arrows) and autolysosomes (green arrows) are indicated. **b** U-87 MG and A172 cells were either left untreated or were treated with TTFields for 24–72 h. CQ (20 µM) was added 4 h before cells were collected. Samples were immunoblotted for LC3 and GAPDH. Upper panel: representative blots. Lower panel: densitometric quantification of immunoblot signal, showing an average of at least three independent experiments (0.01 < *P < 0.05, Student’s *t*-test). **c** U-87 MG and A172 cells were either left untreated or were treated with TTFields for 48 h. CQ (20 µM) was added at the last 3 h of treatment and cells were fixed and stained with anti-LC3 Ab (green) and DAPI (blue). Upper panel: representative images. Original magnifications: × 40. Lower panel: quantification of LC3 intensity, presented as average intensity per cell (**P < 0.01, Student’s *t*-test). **d** U-87 MG cells were either left untreated or were treated with TTFields (48 h) or with vinblastin 25 nM (0.5 h). CQ (20 µM) was added at the last 3 h of treatment and cells were fixed and stained with anti-LC3 (green), LAMP1 (red), and DAPI (blue) (upper panel). Arrowheads indicate the strongest colocalization staining in each cell. Intensity histograms of LAMP1 and LC3 fluorescent signal calculated from the region of interest indicated by the white bar (lower panel). Representative images are shown

The accumulation of autophagosomes, as measured by LC3-II levels, may indicate sheer upregulation of autophagy or it may reflect reduced autophagosome turnover due to defects in autophagosome transport and autophagosome–lysosome fusion. Electron microscopy (EM) observation of ultra-structures revealed membrane-bound vesicles containing cytosolic materials or organelles, and the presence of degradative autophagic vacuoles containing partially degraded material, which was more abundant following TTFields application, indicating the existence of autophagic structures and an effective fusion process (Fig. 3a; blue and green arrows).

To further validate these observations, Chloroquine (CQ; a known inhibitor of lysosomal degradation) was added 3–4 h before treatment end. The addition of CQ, which prevents LC3-II degradation and release back to the cytoplasm, resulted in a significant increase in the LC3-II levels in cells treated with TTFields relative to control cells, as shown by immunoblotting (Fig. 3b, Supplementary Figure 1), immunofluorescent staining for quantification of LC3 puncta (Fig. 3c), as well as autophagic vacuoles accumulation using EM (supplementary figure 2).

Direct evidence for normal autophagosome-to-lysosome fusion under TTFields application was obtained using immunofluorescent staining of LC3 foci with the lysosomal marker LAMP1. Unlike vinblastine, a known inhibitor of autophagosome trafficking, which led to dispersed localization of the two markers, TTFields application did not perturb LC3 and LAMP1 colocalization (Fig. 3d).

Taken together, these results demonstrate that TTFields application lead to an increase in the autophagic flux in glioma cell lines with no substantial influence on the fusion degradation steps.

### Stress response in daughter cells produced under TTFields application upregulate autophagy

**Fig. 4:**
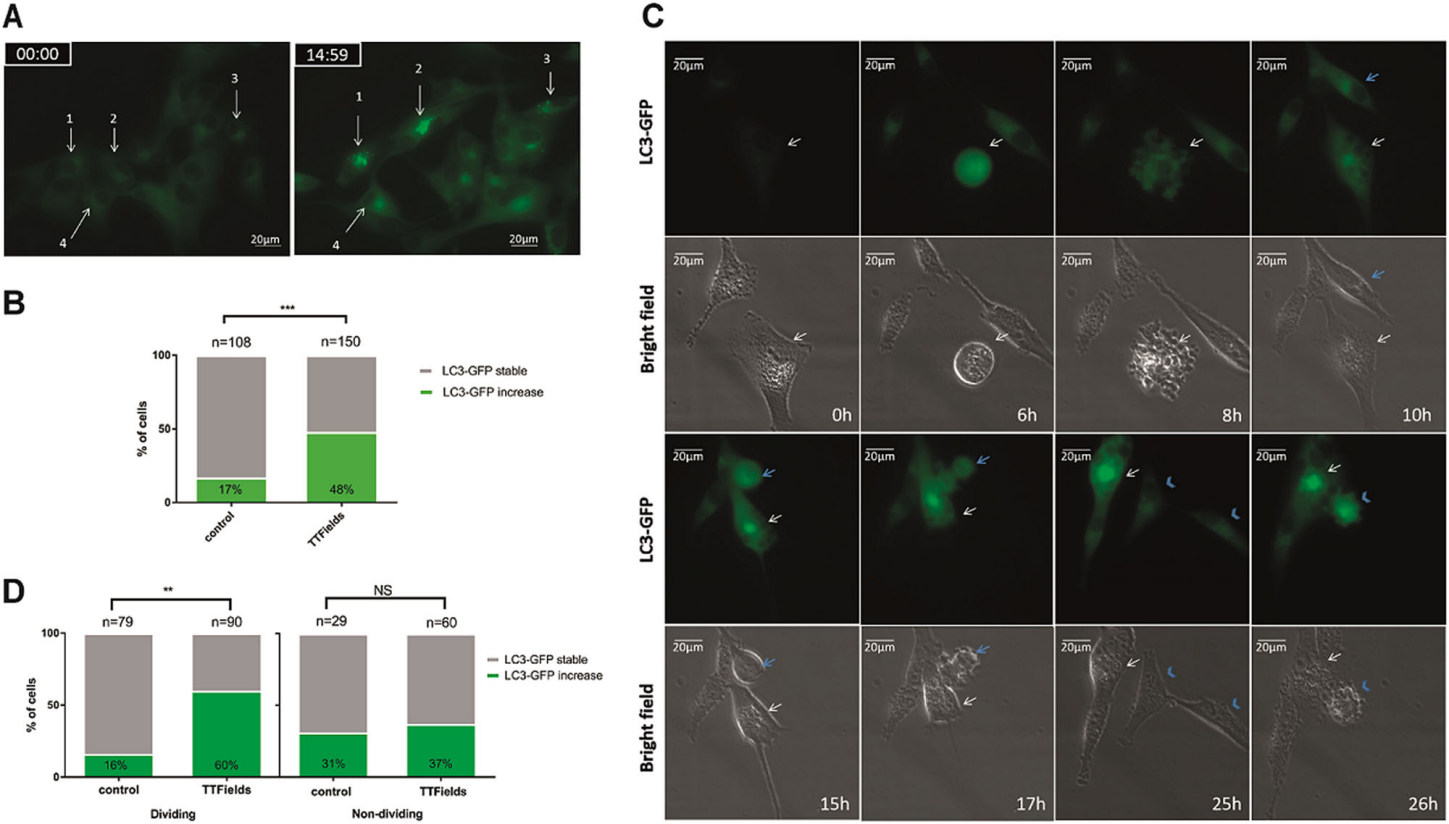
Cells which divided under TTFields application exhibit stress resulting in upregulation of autophagy. Time-lapse microscopy of TTFields-treated (26 h) U-87 MG cells, stably expressing LC3-GFP. **a** Images of representative location in the plate show increase in LC3-GFP punctate signal (green) following first 15 h of TTFields application. Numbered arrows (1–4) indicate the same cell at two different time points (0 and 15 h). **b** Quantification of cells demonstrating increased LC3-GFP signal in control and treatment groups is presented as percent of total cells analyzed. Summary of total amount of cells of three independent experiments (***P < 0.001, *χ*^2^-test). **c** Representative fields of view acquired in time laps microscopy during TTFields treatment. Arrows indicate cells entering mitosis and arrowheads denote the resulting daughter cells. **d** Single-cell analysis of untreated (*n* = 108) and TTFields-treated (*n* = 150) cells, summarized observations from three independent experiments. Cells were monitored for mitotic events and increased LC3-GFP punctate signal (**P < 0.01, NS nonsignificant, *χ*^2^-test)

Abnormal mitosis following TTFields application results in different cell fates including the formation of aneuploid daughter cells^7,8^. Aneuploidy is associated with the activation of regulators of autophagic and lysosomal gene expression^27,28^. To explore whether autophagy was more prominent in cells that divided during TTFields application, we analyzed autophagosome dynamics within single cells using U-87 MG cell line stably expressing LC3 protein fused with green fluorescent protein (GFP). We utilized time-lapse microscopy to monitor the mitotic index, duration of mitosis, and autophagosomes formation in cells during TTFields application. We found that the mitotic index in TTFields-treated cells was somewhat lower than in control group (60% and 73% of cell population, respectively), and that the duration of mitosis was longer in treated cells than in control cells (1.5 h vs. 1 h, respectively, *p* < 0.05) (Supplementary Figure 3). These results are in accordance with previously published data, which demonstrated increased rates of mitotic catastrophe during TTFields application^8,9^. A robust increase in LC3-GFP puncta fluorescence was revealed in 51% of TTFields-treated cells relative to 17% in untreated cells (Fig. 4a, b). Through the analysis of multiple cells, we were able to identify mitotic events within a population and track autophagosome dynamics in the resulting daughter cells (Fig. 4c). Upregulation in LC3-GFP signal was detected in cells that exhibited visible signs of abnormal mitosis such as polyploid nucleus following slippage events (Fig. 4c, white arrow). An increased LC3-GFP signal was also detected in cells that had completed cytokinesis, but where the resulting daughter cells showed abnormal morphology (Fig. 4c, blue arrowhead). Most of the cells that divided during TTFields application showed increased LC3-GFP puncta (60%) relative to 16% of the dividing cells in the untreated culture (Fig. 4d, left). Percentage of cells that did not undergo mitosis, but showed increased LC3-GFP signal, was similar in control and TTFields group (Fig. 4d right). We conclude that the induction of autophagy following TTFields treatment is a consequence of aberrant mitosis.

### TTFields-induced autophagy is mediated by AMPK activation

**Fig. 5:**
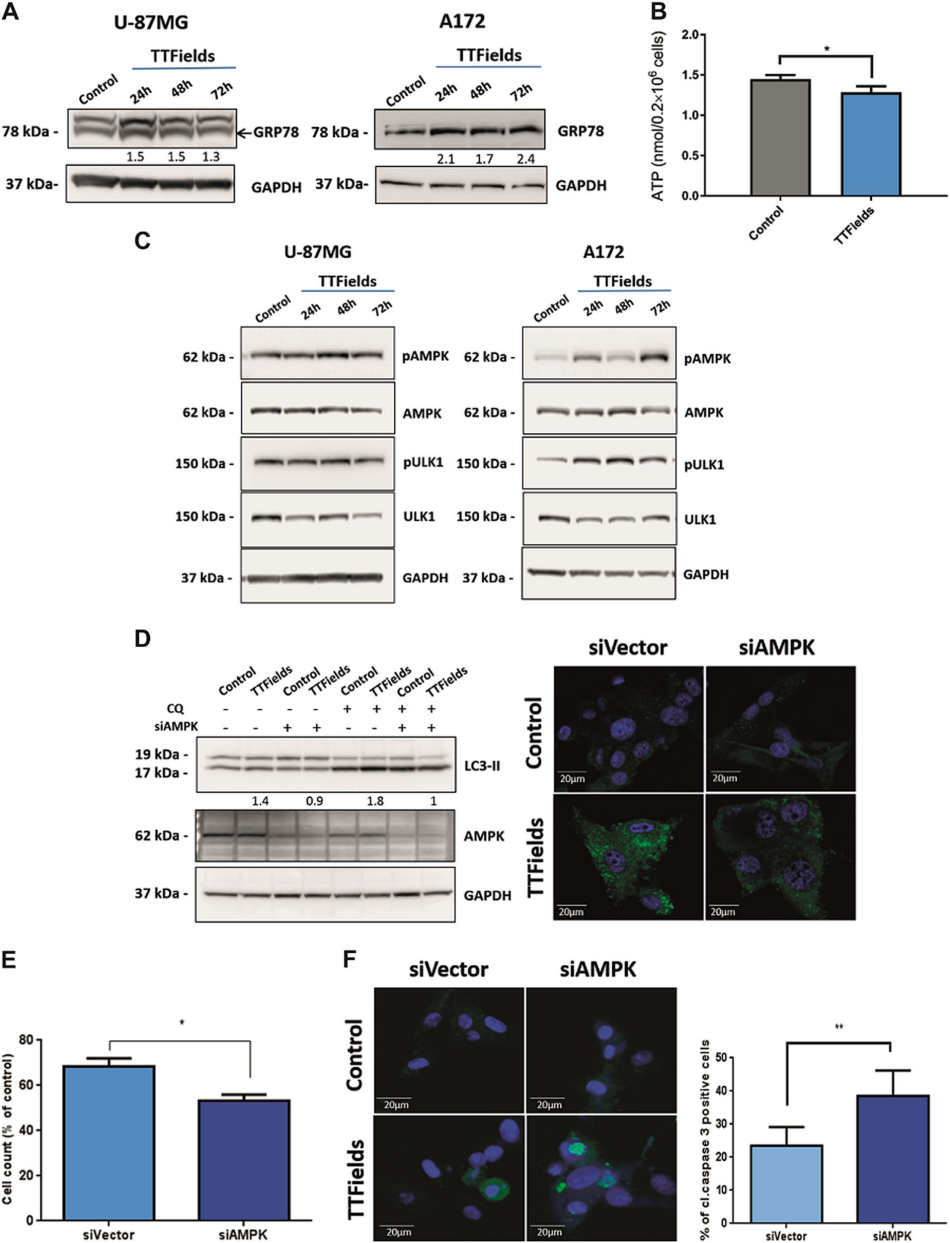
Induction of autophagy by TTFields is AMPK dependent. **a** U-87 MG and A172 cells were either left untreated or were treated with TTFields for indicated time points. Immunoblot analysis of GFP78 protein. Numeric values represent the fold increase in GRP78 signal, normalized to loading control (GAPDH), relative to untreated control. **b** Quantification of intracellular ATP levels in U-87 MG cells either left untreated or treated with TTFields for 72 h.The results are presented as average ATP concentration (nmol/2 × 10^6^ cells) from three independent experiments (*P < 0.01, Student’s *t*-test). **c** U-87 MG and A172 cells were either left untreated or were treated with TTFields for indicated time points. Immunoblot analysis of pAMPK and pULK1proteins. GAPDH was used as loading control. (5D-F) U-87 MG cells were transfected with AMPK-targeting siRNA (siAMPK) or with siRNA sham vector (siVector), and were incubated for 48 h with or without TTFields application. CQ 20 µM was added for the last 4 h of the treatment where indicated. **d** (left panel) Immunoblot analysis of LC3 and AMPK. Numeric values represent the fold-change in LC3-II signal, normalized to GAPDH signal, relative to respective control. **d** (right panel) CQ-treated cells were fixed and stained for LC3 (green) and DAPI (blue), original magnifications: × 40. **e** Cell count of siAMPK- or siVector-expressing cells after the TTFields treatment. (0.01 < *P < 0.05, Student’s *t*-test, *n* = 3). **f** siVector- and siAMPK-transfected U-87 MG cells were either left untreated or were treated with TTFields for 48 h. Cells were then fixed and stained for cleaved caspase-3 (green) and DAPI (blue) (left panel). Images from each treatment were analyzed manually and the fraction of cleaved caspase-3-positive cells was calculated for at least 200 cells from each group (right panel) (**P < 0.01, Student’s *t*-test, *n* = 2)

Aneuploidy-induced proteomic changes may generate proteotoxic stress, which is characterized by the engagement of protein degradation and folding pathways accompanied by additional energy requirements as reflected by low intracellular ATP levels^29,30^. We detected increased levels of GRP78 protein, a common marker of the endoplasmic reticulum unfolded protein response, in both A172 and U-87 MG cells treated with TTFields (Fig. 5a). In addition, intracellular ATP levels were found to be reduced by 18% (*p* < 0.05) in treated cells (~1.2 nmol for 0.2 × 10^6^ cells) relative to untreated (~1.5 nmol for 0.2 × 10^6^ cells) (Fig. 5b).

AMPK, a cardinal cellular energy sensor, is activated by various metabolic stresses and acts as a master positive regulator of autophagy by directly activating of the mammalian autophagy initiating kinase, ULK1^31,32^. Using immunoblot assay, we observed significantly elevated levels of phosphorylated AMPK in U-87 MG and A172 glioma cell lines treated with TTFields at the indicated time points (Fig. 5c, Supplementary Figure 4A). A sequential ULK1 activation indicated by Ser 317 phosphorylation (AMPK-specific site) was also noted (Fig. 5c, Supplementary Figure 4B).

Silencing of AMPK in U-87 MG cells, using small interfering RNAs (siRNAs; siAMPK) resulted in 80% reduction in AMPK protein levels and abrogated the increase in LC3-II levels in response to 48 h TTFields application (Fig. 5d). No changes in LC3-II protein level were observed even after the addition of CQ. Cells transfected with sham vector demonstrated elevated levels of LC3-II after treatment with TTFields similar to U-87 MG wild-type cells (Fig. 5d left panel). Further validation using immunofluorescent staining revealed reduced prevalence of LC3 puncta in siAMPK-transfected cells relative to cells transfected with sham vector following 48 h of TTFields application (Fig. 5d right panel).

TTFields application for 48 h led to a significant reduction in the number of siAMPK cells relative to sham vector transfected cells (47% and 32% reduction in siAMPK and siVector, respectively) (Fig. 5e). The treated siAMPK cells demonstrated elevated levels of cleaved caspase-3 as seen by immunofluorescent staining (Fig. 5f).

Collectively, these results indicate that TTFields’ upregulated autophagy is mediated through the AMPK pathway, and that inhibition of this pathway rendered the cells more susceptible to treatment.

### Inhibition of autophagy enhances TTFields efficacy

**Fig. 6:**
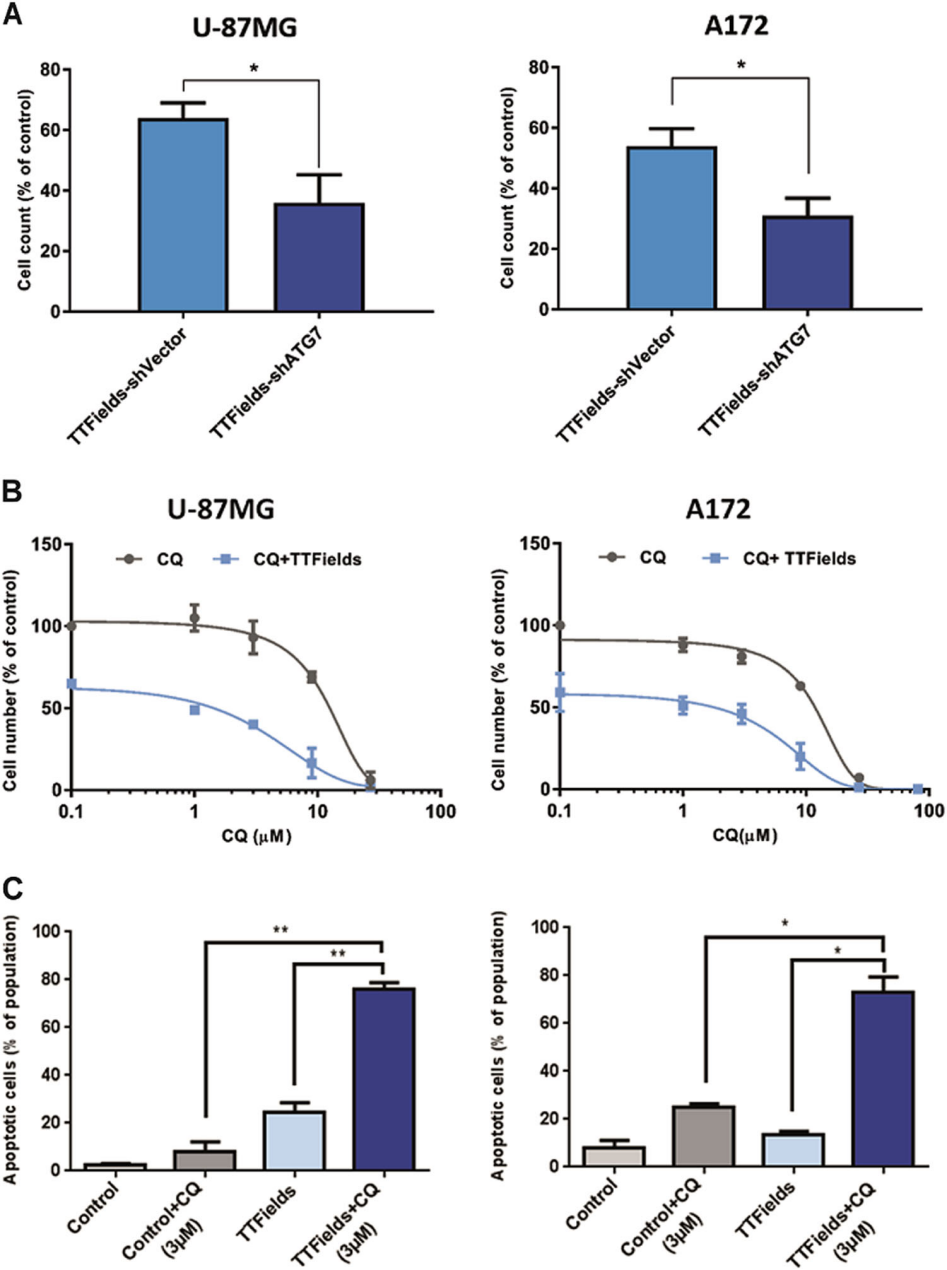
Autophagy inhibition triggers apoptosis and results in increased TTFields efficacy in glioma cells. **a** U-87 MG and A172 were infected with lentiviral particle containing shATG7 or sham vector (shVector). Cells were then either left untreated or were treated with TTFields for 72 h and enumerated by flow cytometry. The data are presented as percent of control. (0.01 < *P < 0.05, Student’s *t*-test, *n* = 3). **b**, **c** U-87 MG and A172 were treated for 72 h with CQ alone (1–27 µM) and in combination with TTFields. **b** Dose–response blots for U-87 MG (left) and A172 (right). **c** Fraction of apoptotic cells as indicated by Annexin V/7-AAD staining in TTFields-treated vs. control cells, with or without CQ (3 μM) (0.01 < *P < 0.05, **P < 0.01, Student’s *t*-test, *n* = 3)

TTFields-enhanced cytotoxic effect following AMPK silencing could be the outcome of an autophagy-independent processes, as AMPK regulates multiple pathways. We utilized a genetic approach to specifically inhibit autophagy in cells using shATG7. Autophagy-related protein 7 (ATG7) is one of the key regulators of autophagosome formation and is responsible for conversion of LC3-I to LC3-II by phosphatidylethanolamine conjugation^33^. For this purpose, we generated lentiviral-mediated shATG7-expressing stable clones in U- 87 MG and A172 cell lines. Inhibition of autophagy led to a significant decrease in cell numbers following 72 h of TTFields application relative to cells expressing sham vector (36% vs. 64% of cell reduction in shVector and shATG7 cells, respectively, in U-87 MG and 46% vs. 62% in A172) (Fig. 6a, Supplementary Figure 5). These results demonstrate that the inhibition of autophagy sensitizes glioma cells to TTFields treatment. Having established the necessity of autophagy for cell survival following TTFields application, we hypothesized that pharmacological targeting of autophagy could potentially provide a promising therapeutic strategy to circumvent resistance to TTFields. To test this, we combined TTFields with CQ in U-87 MG and A172 cells. We found that the combination treatment resulted in a significant reduction in cell numbers. Of note, this improvement in efficacy was observed even at low CQ concentrations (3 µM), whereas CQ monotherapy had no effect on cell counts (Fig. 6b). Flow cytometry analysis revealed higher levels of apoptosis (75%) in cells treated with TTFields in combination with low dose CQ (3 µM), whereas CQ monotherapy resulted in only 25% increase in apoptosis in A172 cells and 8% in U-87 MG cells (Fig. 6c). Overall, these findings suggest that glioma cells upregulate autophagy as a resistance mechanism to TTFields, and that pharmacological inhibition of autophagy circumvents this resistance and enhances the anti-mitotic effects of TTFields.

## Discussion

**Fig. 7:**
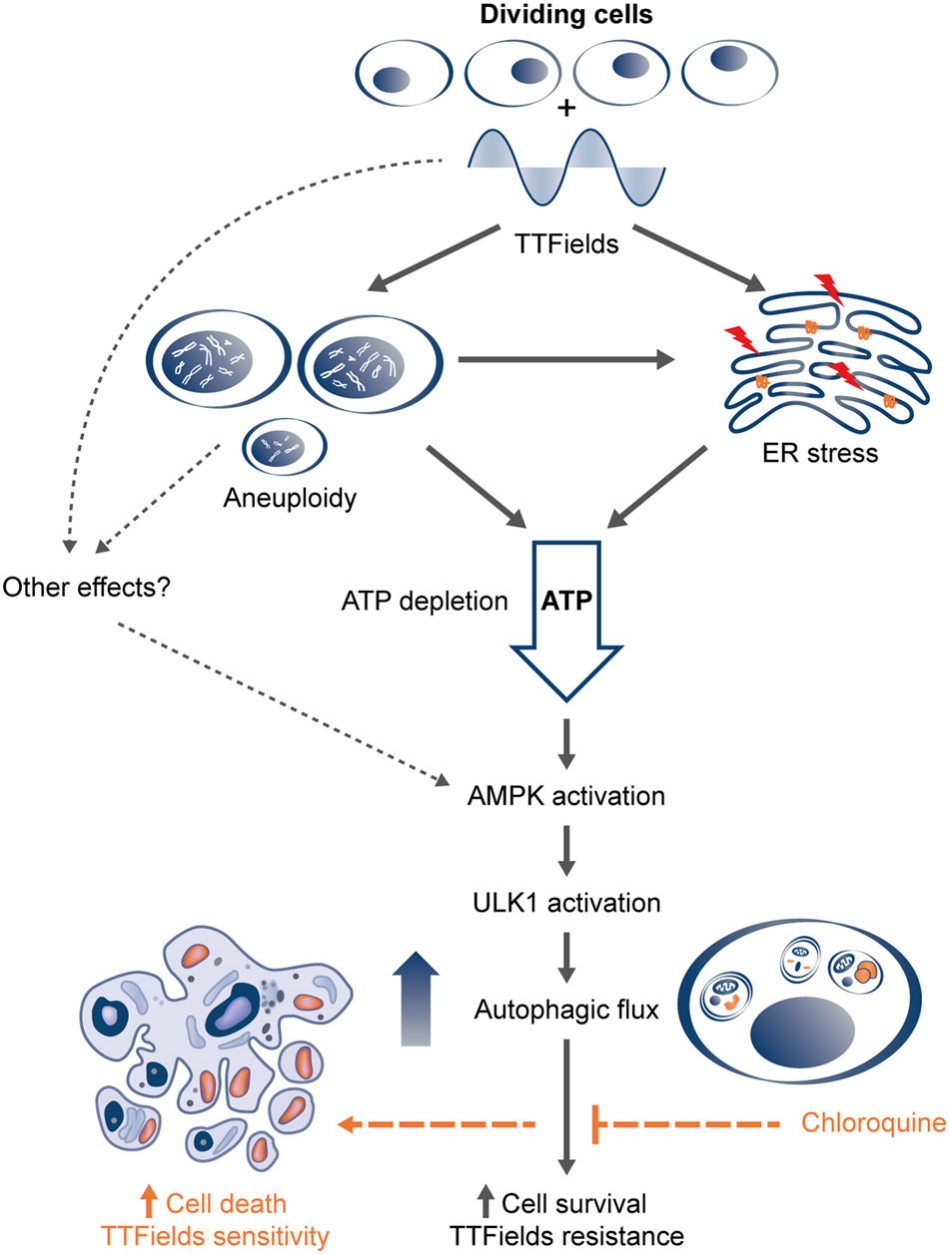
TTFields induce upregulation of autophagy in an AMPK-dependent manner in dividing cells. TTFields application to dividing cancer cells leads to disruption of the mitotic spindle and abnormal chromosome segregation, which mostly trigger different forms of cell death. Aneuploidy, ER stress, and low intracellular ATP levels are characteristics of the surviving daughter cells. These features, and potentially other AMPK-activating cascades, trigger AMPK and sequential ULK1 activation, which results in increased autophagic flux. High autophagy exerts cellular survival under TTFields treatment, thus acting as a resistance mechanism. Addition of CQ increases cell death under treatment and therefore enhances TTFields efficacy

TTFields therapy is a physical treatment modality that has been demonstrated to improve both progression-free and overall survival in glioblastoma patients when added to standard maintenance temozolomide chemotherapy^4^. Previous studies have demonstrated that TTFields exert an anti-mitotic effect by decreasing the fraction of polymerized tubulin during mitosis thus hampering normal spindle organization and producing severe structural mitotic deformities. These may lead to chromosome aneuploidy in the resulting daughter cells^8^. How cells survive the stress inflicted by TTFields is poorly understood. In this study, we demonstrate that daughter cells surviving TTFields application upregulate autophagy in response to proteotoxic stress, thereby promoting resistance to treatment (Fig. 7).

As TTFields act as a microtubule depolymerization agent, and as microtubules have an important role in both autophagosome transport and autophagosome–lysosome fusion, we speculated that the observed increase in autophagosome accumulation may result from interference in these processes, as demonstrated in cells treated with vinblastine (a known microtubule depolymerization agents)^34^. However, our results demonstrate successful autophagosomes-to-lysosome fusion under TTFields application, suggesting that such perturbation to the autophagy process is not inflicted by TTFields.

It is important to better understand how TTFields application promote autophagy in treated cells. It has been proposed that cells respond to aneuploidy by engaging proteotoxic stress response, which includes upregulation of pathways leading to the degradation of cellular constituents and protein folding^30^. Specifically, upregulation of autophagy was observed consistently in aneuploid cells^28^. We exploited single-cell analysis to show for the first time that TTFields application specifically triggers autophagy in the progeny cells that divided during treatment. This finding suggests that TTFields enhance autophagy in glioma cells as part of a cellular response to aberrant mitosis, with aneuploidy having a crucial role. Aneuploidy-induced proteomic change was previously shown to generate proteotoxic stress accompanied by additional energy requirements as reflected by low intracellular ATP levels^29,30^. We demonstrate that following TTFields application, there is an increase in endoplasmic reticulum stress and reduced levels of intracellular ATP. Our data indicate that TTFields-induced autophagy is mediated and dependent on AMPK activity, which is in line with the observed proteotoxic stress response and reduced ATP intracellular levels, as AMPK is activated by low-energy status. Therefore, we propose that autophagy is triggered via AMPK activation in response to aneuploid status following cell division under TTFields application (Fig. 7).

It is noteworthy that Karanam et al.^11^ have recently shown that TTFields can potentially induce replication stress and reduction of BRCA1 pathway proteins, eventually leading to genotoxic stress.

Therefore, in addition to aneuploidy, AMPK-dependent induction of autophagy can also be potentially triggered by such genotoxic stress. For example, the serine/threonine kinase ATM, which is a major sensor of DNA double-strand breaks, can activate AMPK, thus leading to induction of autophagy^35,36^. Another example is the DNA damage repair enzyme PARP1, which is associated with elevated AMP levels that activate AMPK^37^. In the present study, we have not examined whether these pathways are activated following TTFields application and future studies are needed to assess the contribution of TTFields-induced genotoxic stress to autophagy.

Induction of autophagy in normal tissue may exert a detrimental effect and lead to autophagic cell death^38,39^. Effective TTFields intensities are present not just in the tumor but also in the surrounding tissues and may potentially affect normal bystander cells. However, both preclinical and clinical evidence did not reveal adverse events in normal tissues. Our findings demonstrate that the upregulation of autophagy by TTFields is restricted to dividing cells, which may explain the relatively lack of safety issues with this modality.

Extensive preclinical evidence for the involvement of autophagy in cancer cell survival has led to a rise in clinical trials, which are underway to evaluate the therapeutic potential of autophagy inhibitors in combination with chemotherapy^18,40,41^. A preclinical glioma model showed that autophagy upregulation in response to temozolomide confers resistance to treatment^42^. Although TTFields differ significantly in their mode of action from chemo and radiotherapy, our data indicate a similar resistance pattern of cells utilizing autophagy to evade lethal effects. Specifically, our results demonstrate that blocking autophagy induced by TTFields by using either genetic or pharmacologic approaches, results in increased treatment efficacy. CQ and its derivative hydroxychloroquine, a broadly used anti-malarial agent, are currently the only clinically available drugs to inhibit autophagy^43^. In our study, combination of TTFields with CQ resulted in decreased cell viability in a dose-dependent manner. Moreover, the presence of low concentration of CQ, which has no effect on cell number as a monotherapy, resulted in high levels of apoptosis when combined with TTFields (Fig. 7). These results indicate increased treatment efficacy for the combined treatment and provide strong rationale for additional in-vivo studies.

Interestingly, a recent study by Silginer et al.^17^ demonstrated that the concomitant use of 3-Methyladenine (3-MA; early-stage inhibitor of autophagy) during TTFields application result in higher cell viability. 3-MA is known to block class I and class III PI3K activity, thus exhibiting dual activity that could lead to either inhibition or upregulation of autophagy^44^. Targeting a complex process such as autophagy may lead to different consequences depending on the exact stage of autophagy being targeted. It has been reported that in treatment regimens utilizing cytotoxic drugs combined with agents that inhibit autophagy at an early stage, the cytotoxic effect was hindered. On the other hand, inhibition at late stages in autophagy (e.g., using bafilomycin or CQ) led to enhanced efficacy of the same treatment as reflected by increased cytotoxic effect, suggesting that accumulation of autolysosomes is necessary for cell death induction^45,46^. Although CQ is primarily acknowledged as blocker of lysosomal degradation, a recent study by Mauthe et al.^47^ demonstrated that CQ can also inflict severe disorganization of the Golgi and endo-lysosomal systems. Therefore, it is imperative to also acknowledge the autophagy-independent effects of CQ and the possibility that some elements of the enhanced treatment efficacy of the combined treatment with CQ may be attributed to other autophagy-unrelated cellular alterations.

The combination of CQ with two additional drugs, 17-allylamino-17-demethoxy-geldanamycin and 5-aminoimidazole-4-carboxamide riboside, had been shown to promote proteotoxic and metabolic stress leading to the induction of apoptosis exclusively in aneuploid cells^27^. Future work is warranted on these and other therapeutic combinations that could leverage the stress response induced by TTFields, to further enhance treatment efficacy.

## Materials and methods

### Cell lines and cultures

All cell lines were obtained from ATCC: MSTO-211H (human biphasic mesothelioma), KLN-205 (murine squamous cell carcinoma), LLC-1 (murine Lewis lung carcinoma), AsPC-1 (pancreatic adenocarcinoma), A172, U-87 MG, LN229 (human glioma cell lines), and F98 (rat glioma). Cells were cultured in Dulbecco’s modified Eagle’s medium (Biological Industries) or RPMI (GIBCO) medium supplemented with 10% fetal bovine serum and antibiotics.

### TTFields application

TTFields were applied to cell cultures using the inovitro™ system (Novocure Ltd) as described^48,49^. Cells were seeded on cover slips at a density of 5000–20,000 cells in 500 µL and treated at predetermined optimal frequencies: MSTO-211H (150 kHz), KLN-205 (150 kHz), LLC-1 (150 kHz), AsPc-1 (150 kHz), A172 (200 kHz), U-87 MG (200 kHz), LN229 (200 kHz), and F98 (200 kHz) at the same nominal intensity (1.75 V/cm RMS). TTFields were applied from two directions, which were changed by 90° every 1 s as previously described^3^. Culture media (2 ml per dish) was replaced every 24 h for all control and treatment dishes.

### Flow cytometry

To assess cellular granularity, cells treated with TTFields for 72 h were analyzed based on their side-scatter values. Evaluation of treatment efficacy was quantitatively determined by cell count after specified treatment duration. The relative number of cells at the end of treatment was expressed as a percentage of untreated control. For detection of apoptosis, cells were double‐stained with fluorescein isothiocyanate‐conjugated Annexin V (MEBCYTO 4700 Apoptosis Kit; MBL) and 7‐Aminoactinomycin D (BioLegend) as per the manufacturer’s instructions. Data acquisition was obtained using iCyt EC800 (Sony Biotechnology) flow cytometer.

### LysoTracker staining

Cells were stained for 80 min in 37 °C with 75 nM LysoTracker probe (Molecular Probes). Images were obtained using upright motorized microscope with × 40/0.75 objective (ZeissAxio Imager Z2) equipped with the Orca R2 camera (Hamamatsu Photonics, Japan).

### Immunocytochemistry

For autophagy assessments, cells were grown on glass cover slips and treated using the inovitro^TM^ system (Novocure, Israel) for 48 h. At the end of the treatment, cells were fixed with ice‐cold methanol for 10 min. The cells were then serum‐blocked and stained with microtubule-associated protein 1 LC3 (rabbit polyclonal, Novus) and LAMP1 antibody (mouse monoclonal, Santa Cruz). Alexa Fluor 488- or 533-conjugated secondary antibody was used (Jackson ImmunoResearch). DNA was stained with the dye 4′,6‐diamidino‐2‐phenylindole (DAPI) (Sigma‐Aldrich) at 0.2 µg/ml for 20 min. Images were collected using a LSM 700 laser scanning confocal system, attached to an upright motorized microscope with × 63/1.40 oil objective (ZeissAxio Imager Z2). To better detect LC3 by immunofluorescence, cells were cultured in the presence of CQ diphosphate (Sigma) (CQ, 20 µM) during the last 3 h of the treatment. CQ was applied to both control and TTFields-treated cells. Colocalization of LAMP1 and LC3 was tested in three individual cells from each treatment using the profile tool in the Zen 2.3. software (Blue edition; Carl Zeiss Microscopy, GmbH). Areas in which at least two LC3 foci were apparent were used for the analysis. For cleaved caspase-3 staining following treatment, cells were fixed with paraformaldehyde (PFA) 4%, permeabilized with 0.3% Triton solution in phosphate-buffered saline (PBS) (× 1), and stained with anti-cleaved caspase-3 antibody (rabbit polyclonal, Cell Signaling). The quantification of intensity of green signal, reflecting amount of LC3-positive puncta, was carried out using ImageJ software and presented as percentage of average intensity per cell normalized to average intensity in untreated cells. The quantification of cleaved caspase-3-positive cells was done manually in a blinded manner. The data were presented as percent of positive stained cells.

### Cell lysates and immunoblotting

Cell extracts were prepared using RIPA lysis buffer containing 150 mM sodium chloride, 1% NP-40, 0.1% SDS, 50 mM Tris pH = 8, supplemented with a cocktail of protease (Complete Mini, Roche), and phosphatase inhibitors (Thermo Scientific). After determining protein concentration (Bradford reagent, Bio-Rad), 30 µg protein were resolved by SDS-polyacrylamide gel electrophoresis under reducing conditions. After electrophoresis, proteins were transferred to polyvinylidene difluoride membrane (Bio-Rad) and probed with the appropriate primary antibody: LC3 (NB600-307) purchased from Novus, pAMPK (2535), AMPK (2793), pULK1 (12753), ULK1 (8054), pP70 (9205s), ATG7 (8558) from Cell Signaling; GAPDH (SC-32233), GRP78 (BiP) (SC-376768) from Santa Cruz; Vinculin (V9131) from Sigma-Aldrich followed by horseradish peroxidase-conjugated secondary antibody (Abcam ab6721, ab97023), Rockland (111-035-144)), and a chemiluminescent substrate (Millipore).

### siRNA-mediated knockdown of Ampk

U-87 MG cells were transfected with siRNA specific for Ampk (SMARTpool: ON-TARGETplus Human PRKAA1 siRNA, Dharmacon) using Lipofectamine (Invitrogen, Life Technologies Corporation) according to the manufacturer’s instruction. Cells were seeded on cover slips in inovitro dishes 24 h before transfection. At the time of the experiment, the cells had reached 50% confluency. The culture medium was replaced with Opti-MEM (Invitrogen) containing 20 nM of siRNA mixed with 1 ml of Dulbecco’s Modified Eagle antibiotics-free medium and 10 µl of RNAi-Lipofectamine 2000. After 48 h, dishes were connected to the inovitro systems and TTFields were applied for 48 h.

### Time-lapse microscopy

U-87 MG cells were transfected with pSELECT-GFP-hLC3 vector (InvivoGen) using Lipofectamine (Invitrogen, Life Technologies Corporation) according to the manufacturer’s instruction. Following transfection, cells were washed and selected for resistance to zeocin (600 µg/ml) (InvivoGene). Stable transfected cells were observed for 24 h using time-lapse series microscopy (ZeissAxio Observer; × 10 objective) either with or without TTFields. TTFields (1.75 V/cm) were applied using the inovitro Live^™^ system (Novocure). Briefly, two pairs of transducer arrays were printed perpendicularly on the outer walls of a cylinder inovitro Live insert composed of high dielectric constant ceramic [lead magnesium niobate–lead titanate]. The transducer arrays were connected to a sinusoidal waveform generator that generated the electric fields in the medium. The orientation of the TTFields were switched 90° every 1 s, thus covering the majority of the orientation axis of cell divisions as previously described by Kirson et al.^3^. Temperature was measured by 2 thermistors (Omega Engineering, Stamford, CT) attached to the ceramic walls. Cells were grown in high Glass Bottom 35 mm µ-Dish (Ibidi, GMBH). The inovitro Live insert was placed inside the glass bottom dish. Image stacks were acquired every 15 min.

### Time-lapse microscopy data analysis

Single cells were followed manually by two independent investigators. Mitotic events were recorded in a blinded manner. Images of cells before and after mitosis were analyzed using ImageJ software (NIH) as described by Veldhoen et al.^50^, and modified to accommodate single-cell analysis. Briefly, puncta were identified by generating surfaces in ImageJ after background subtraction (Rolling Ball Background, rolling = 20) and setting of identical threshold to all images. The fluorescence intensity localized to all puncta in each cell after mitosis were divided by the fluorescence intensity measured in the same cell before mitosis. A threshold value of twofold increase in LC3-GFP fluorescence intensity relative to the initial state of the cell was defined as “increased.” For non-dividing cells, images from the beginning and the end of treatment were analyzed and compared in the same manner.

### Electron microscopy

A172 and U-87 MG cells, control or treated with TTFields for 48 h, were fixed in PFA (3%) + glutaraldehyde 2.5% + 0.1 M cacodylate buffer + 5 mM CaCl + 3% sucrose and further processed for blocking and section preparation. Thin sections (70 nm) were coated with carbon and visualized using Zeiss Ultra-Plus FEG-SEM equipped with transmission electron detector, at acceleration voltage of 30 kV. In addition, U-87 MG cells control or treated, in the presence of CQ (1 µM, 24 h) (Sigma-Aldrich), were fixed as described and were visualized using JEOL JEM-1011 TEM. Count of autophagic vacuoles was performed manually in a blinded manner.

### shRNA lentiviral infection

Human ATG7 short hairpin RNA (shRNA) and non-silencing-GIPZ lentiviral shRNAmir control were purchased from Dharmacon. shATG7 containing viral particles were produced using LENTI-Smart kit (InvivoGen), according to the manufacturer’s instruction, in 293T cells. U-87 MG and A172 cells were infected with lentiviral particles; 24 h after infection, cells were washed and selected for puromycin (Sigma) (2 µg/ml) resistance for 3 days. ATG7 protein levels in stable culture were further validated in western blot analysis with specific anti ATG7 (rabbit monoclonal, Cell Signaling) antibody.

### ATP measurement

ATP intracellular levels were measured with colorimetric ATP assay kit (Abcam) according to the manufacturer’s instructions. After 48 h of treatment, cells were collected and 1 × 10^6^ cells from each sample were resuspended in ATP lysis buffer.  Trichloroacetic acid (100%) was used for sample deproteinizing followed by neutralization step KOH (1 M). ATP reaction mix and background control (50 µL) was added to the wells and incubated for 30 min in dark, followed by an absorbance measurement at 550 nm using an ELISA reader (infinite F200, Tecan).

### In-vivo application of TTFields

All animal studies were approved by the Novocure Internal Animal Care Committee in accordance with the Technion‐Israel Institute of Technology guidelines for the care of laboratory animals. Twelve-week-old Male Fischer rats (Harlan Laboratories, Israel) were inoculated stereotactically into the subcortical white matter in the right hemisphere with glioma (F98) cells (1 × 10^4^), as previously described^2^.

Rats were allowed to recuperate for 7 days before treatment initiation. Application of TTFields (200 kHz) to the rat brain was initiated 7 days after intracranial tumor inoculation and was maintained for 7 days. Two pairs of electrodes, each composed of two disks with a radius of 3 mm, were attached to the rat skull in dorsolateral and left–right positions generating two different field directions. The capacitance of each disk was about 30 nF. Each disk contained a thermistor in order to allow for constant temperature monitoring. The current source output was switched, every 1 s, between the two electrodes. Control rats were treated by means of sham electrodes, which were geometrically matched to the TTFields group. The Sham heat electrodes produced equal temperature changes to those produced by the field electrodes by means of a heating resistor incorporated within them. Each rat was placed inside a separate cage and the electrodes were connected to the NovoTTF-100A^TM^ device. Rats were checked twice daily for their physical condition. At the end of treatment, the rats were killed and the tumors were removed.

### Immunohistochemistry

For immunofluorescent staining, paraffin-embedded tumor sections were deparaffinized with HistoChoice (Sigma-Aldrich) and rehydrated with graded alcohol treatments. Antigen retrieval was carried out by microwave treatment for 22 min in citrate buffer (pH 6.0). Sections were blocked in 10% normal goat serum in PBS and incubated overnight with primary antibody (LC3, Novus) following secondary antibody (Alexa Flour 488, Jackson ImmunoResearch) incubation and DAPI for nuclei counterstaining. The whole slide image was collected using Automatic slide scanner 250 Flash (3DHISTECH). The quantification of intensity of green signal, reflecting amount of LC3 staining, was carried out using CaseViewer following ImageJ software. Three different areas of similar size from each image were chosen in a blinded manner to be analyzed by ImagJ software. Average green intensity per image was calculated. The data are presented as average intensity relative to control in each set of staining.

### Statistical analysis

Data are presented as means ± SE. Statistical significance was analyzed by the two-tailed Student’s *t*-test. *χ*^2^-analysis was applied to determine significant relationship between mitosis and TTFields treatment (GraphPad Prism 6 utility software). Values of *P* < 0.05 were considered significant. All experiments were repeated at least three times with similar results.

## Electronic supplementary material

Supplementary figure legends

Supplementary Figure 1: TTFields application induce an increase in autophagic flux

Supplementary Figure 2: Electron micrographs reveal increased levels of autophagosome like structures in U-87 MG cells following TTFields application

Supplementary Figure 3: TTFields disrupt mitosis in U-87 MG cells

Supplementary Figure 4: Induction of autophagy by TTFields is AMPK dependent

Supplementary Figure 5: Atg7 expression levels
