# Supplementary material for: AMPK-dependent autophagy upregulation serves as a survival mechanism in response to Tumor Treating Fields (TTFields)
Source: Cell Death Dis. 2018 Oct 19;9(11):1074. doi: 10.1038/s41419-018-1085-9 (PMC6195570; doi:10.1038/s41419-018-1085-9)
Supplement: Supplementary file 1 — Supplementary figure legends [file 41419_2018_1085_MOESM1_ESM.docx]

**AMPK-dependent autophagy upregulation serves as a survival mechanism in response to Tumor Treating Fields (TTFields)**

Anna Shteingauz*, Yaara Porat*, Tali Voloshin, Rosa S. Schneiderman, Mijal Munster, Einav Zeevi, Noa Kaynan, Karnit Gotlib, Moshe Giladi, Eilon D. Kirson, Uri Weinberg, Adrian Kinzel, Yoram Palti.

**Supplementary Figures**

**Sup. Fig 1: TTFields application induce an increase in autophagic flux**

U-87 MG cells were either left untreated, or treated with TTFields for 24- 72h. CQ (20 µM) was added 4h before cells were collected. Samples were immunoblotted for LC3 and GAPDH. Representative blots are shown.

**Sup. Fig 2: Electron micrographs reveal increased levels of autophagosome like structures in U-87 MG cells following TTFields application**

(1A) TEM micrographs of U-87 MG untreated cells (bottom) or treated with TTFields (top left) for 24h (X8000 magnification), in presence of CQ. Arrows indicate representative autophagic structures (x40000 magnification). (1B) Quantification of autophagic vacuoles per cell of control (n=8) and TTFields treated (n=6) cells (***p < 0.001, student's t-test).

**Sup. Fig 3: TTFields disrupt mitosis in U-87 MG cells**

U-87 MG cells stably expressing LC3-GFP were either left untreated, or treated with TTFields for 24h. Mitotic duration was calculated for each group by quantitative analysis of time-lapse microscopy (n=150 treated and n=108 control cells) (0.01<*P < 0.05, student's t-test).

**Sup. Fig 4:** **Induction of autophagy by TTFields is AMPK dependent**

Densitometric quantification of immunoblot signal of phosphorylated protein to total protein ratio for AMPK (3A) and ULK1 (3B) (0.01<*P < 0.05, **P<0.01, ***P<0.001, from corresponding control, student's t-test, n=3).

**Sup. Fig** **5: Atg7 expression levels**

Atg7 expression in U-87 MG (left panel) and A172 (right panel) after shRNA lentiviaral transfection was validated by immunoblotting of cell lysates and densitometric quantification of Atg7 signal relative to GAPDH as loading control in every experiment. Representative blots are shown.
