# Supplementary figures and images for: AMPK-dependent autophagy upregulation serves as a survival mechanism in response to Tumor Treating Fields (TTFields)
Source: Cell Death Dis. 2018 Oct 19;9(11):1074. doi: 10.1038/s41419-018-1085-9 (PMC6195570; doi:10.1038/s41419-018-1085-9)

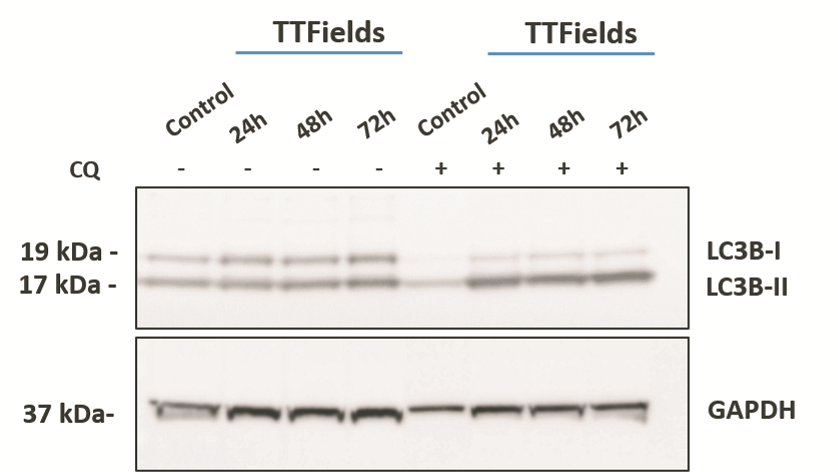

Supplement: Supplementary file 2 — Supplementary Figure 1: TTFields application induce an increase in autophagic flux [file 41419_2018_1085_MOESM2_ESM.tif]

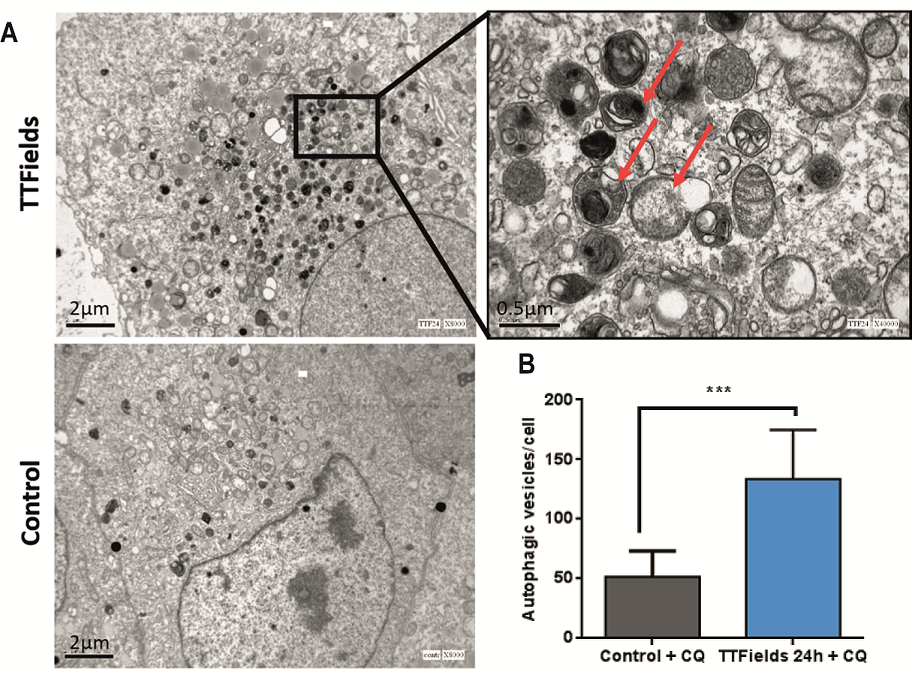

Supplement: Supplementary file 3 — Supplementary Figure 2: Electron micrographs reveal increased levels of autophagosome like structures in U-87 MG cells following TTFields application [file 41419_2018_1085_MOESM3_ESM.tif]

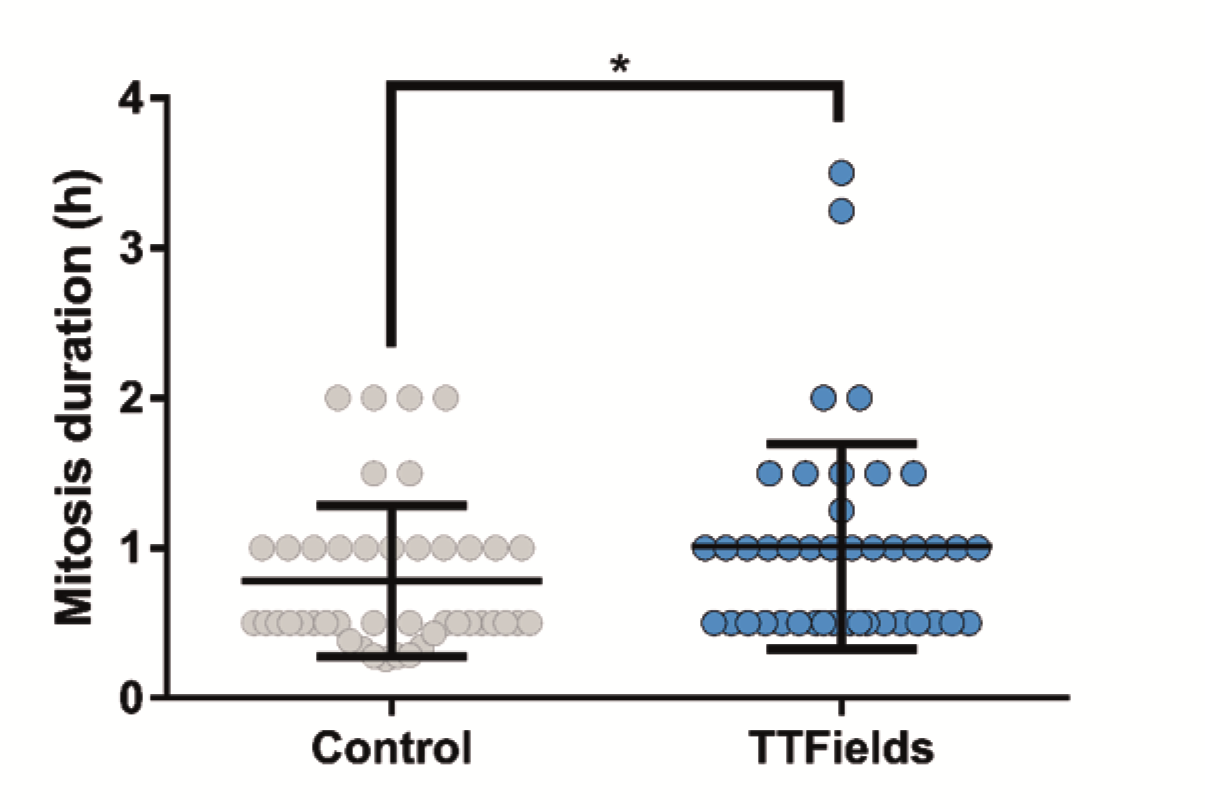

Supplement: Supplementary file 4 — Supplementary Figure 3: TTFields disrupt mitosis in U-87 MG cells [file 41419_2018_1085_MOESM4_ESM.tif]

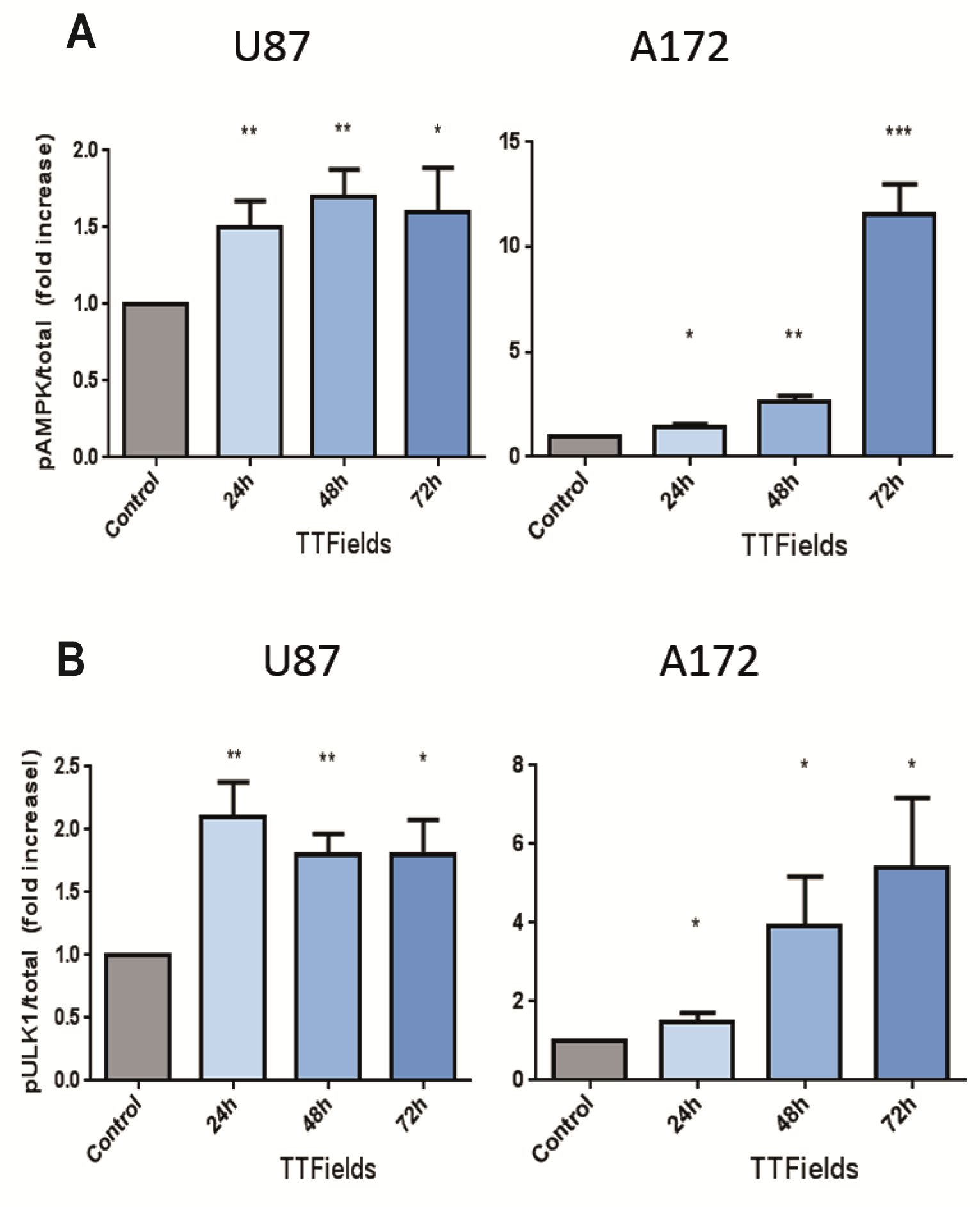

Supplement: Supplementary file 5 — Supplementary Figure 4: Induction of autophagy by TTFields is AMPK dependent [file 41419_2018_1085_MOESM5_ESM.tif]

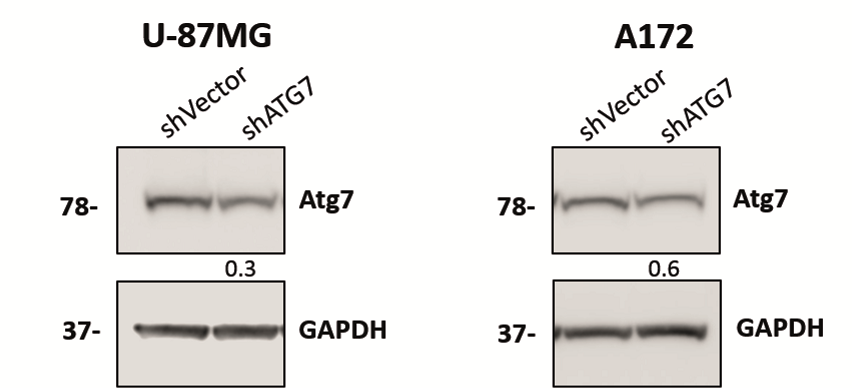

Supplement: Supplementary file 6 — Supplementary Figure 5: Atg7 expression levels [file 41419_2018_1085_MOESM6_ESM.tif]
